# Classification of Hyperspectral Images of Explosive Fragments Based on Spatial–Spectral Combination

**DOI:** 10.3390/s24227131

**Published:** 2024-11-06

**Authors:** Donge Zhao, Peiyun Yu, Feng Guo, Xuefeng Yang, Yayun Ma, Changli Wang, Kang Li, Wenbo Chu, Bin Zhang

**Affiliations:** 1 State Key Laboratory of Dynamic Measurement Technology, North University of China, Taiyuan 030051, China; 2 School of Information and Communication Engineering, North University of China, Taiyuan 030051, China; b20240511@st.nuc.edu.cn (P.Y.); s2005063@st.nuc.edu.cn (F.G.); yxf768@nuc.edu.cn (X.Y.); mayayun@nuc.edu.cn (Y.M.); 20160080@nuc.edu.cn (B.Z.); 3 Northwest Institute of Nuclear Technology, Xi’an 710024, China; wangchangli@nint.ac.cn (C.W.); likang@nint.ac.cn (K.L.); 4 College of Mechanical and Electrical Engineering, North University of China, Taiyuan 030051, China; 20210008@nuc.edu.cn

**Keywords:** hyperspectral image classification, fragments detection, convolutional neural network–bidirectional long short-term memory network, u-shaped network, spatial–spectral combination

## Abstract

The identification and recovery of explosive fragments can provide a reference for the evaluation of explosive power and the design of explosion-proof measures. At present, fragment detection usually uses a few bands in the visible light or infrared bands for imaging, without fully utilizing multi-band spectral information. Hyperspectral imaging has high spectral resolution and can provide multidimensional reference information for the fragments to be classified. Therefore, this article proposed a spatial–spectral joint method for explosive fragment classification by combining hyperspectral imaging technology. In a laboratory environment, this article collected hyperspectral images of explosion fragments scattered in simulated scenes. In order to extract effective features from redundant spectral information and improve classification accuracy, this paper adopted a classification framework based on deep learning. This framework used a convolutional neural network–bidirectional long short-term memory network (CNN-BiLSTM) as the spectral information classification model and a U-shaped network (U-Net) as the spatial segmentation model. The experimental results showed that the overall accuracy exceeds 95.2%. The analysis results indicated that the method of spatial–spectral combination can accurately identify explosive fragment targets. It validated the feasibility of using hyperspectral imaging for explosive fragment classification in laboratory environments. Due to the complex environment of the actual explosion site, this study still needs to be validated in outdoor environments. Our next step is to use airborne hyperspectral imaging to identify explosive fragments in outdoor environments.

## 1. Introduction

The performance parameters of fragments primarily involve the number [1], material composition, and velocity. Identifying explosive fragments is crucial for assessing explosive power and designing effective explosion-proof measures. Currently, research primarily focuses on evaluating target damage effectiveness [2] and implementing optical imaging techniques [3]. The dispersion [4], shape [5], average quality [6], and other characteristics of explosive fragments hold significant importance in performance evaluations. However, existing fragment detection methods only provide one-dimensional information about fragments, such as mass, spatial details, and spectral information, typically captured using a single band, such as visible or infrared. These methods overlook the varying degrees of light absorption at different wavelengths by fragments and background materials, failing to extract the multidimensional information of the fragments. Consequently, incorporating multidimensional information for fragment analysis is essential for enhancing their structure and performance.

Hyperspectral imaging technology combines the advantages of traditional imaging technology and spectral technology [7]. It can obtain the spatial information and the spectral information of continuous bands in the images [8]. Hyperspectral images can provide reference images for spatial positions and provide multi-dimensional information for target classification. In the realm of object detection, hyperspectral imaging technology offers substantial advantages in the enhancement of classification accuracy [9]. Over the course of several decades, this technology has found applications in civilian sectors, including agricultural production [10], landscape characterization [11], environmental monitoring [12], and food ingredient identification [13]. In military domains, hyperspectral detection technology has been successfully employed in the recognition of maritime and shallow-water targets [14], battlefield camouflage [15], and land targets [16], showcasing its extensive range of potential applications.

The small size and irregular shape of the generated fragments pose challenges for conventional imaging methods in accurately identifying ground fragments. The fragments and background materials have different molecular structures, leading to variations in the reflection characteristics of light across different wavelengths. On the other hand, hyperspectral images provide a contiguous spectral signature for each pixel, reflecting the physical and chemical properties of objects [17]. Therefore, the method of hyperspectral imaging can capture more detailed information under different spectra [18], making it highly suitable for distinguishing between different fragments and background materials. The recognition of iron fragments was studied to verify the feasibility of hyperspectral detection methods for iron fragment recognition based on traditional methods [19].

In comparison to traditional classification methods, deep learning offers the advantage of automated feature extraction and selection within the model, eliminating the need for manual feature construction [20]. At present, various methods of deep learning have been proposed for the classification of hyperspectral images [18,21]. Compared with other deep-learning methods, convolutional neural networks (CNN) have extensive applications in hyperspectral image classification due to their excellent spectral feature representation. Hu first used the CNN in hyperspectral image classification with only a one-dimensional (1D) convolution kernel and focused on the spectral features of hyperspectral imagery [22]. Despite CNNs having achieved promising results in most cases [23,24], they still encounter the following limitations:

(1) CNN is a vector-based approach that treats the input of hyperspectral images as a collection of pixel vectors [25]. The equal treatment of all channels can lead to a CNN being incredibly insensitive to the spectral sequence information [26]. The results are that the feature maps cannot effectively represent the spectral sequence information from hyperspectral images;
(2) A 1D CNN can only consider the spectral information of hyperspectral images and ignores the spatial relationships between pixels.

However, the spectral information of the sample points in hyperspectral images is typical sequence data. The change in reflectivity intensity of adjacent bands usually leads to a change in the overall trend of reflectivity. While CNNs are known for their automatic feature-extraction capabilities [27], recurrent neural networks (RNNs) are more adept at handling sequential data [28]. Specifically, bidirectional long short-term memory networks (BiLSTM), a type of RNN, are well-suited for processing time series data and effectively capturing the relationships between the current data and their preceding or subsequent data points [29]. Taking advantage of both CNNs and RNNs, we adopted a combined model called a convolutional neural network–bidirectional long short-term memory network (CNN-BiLSTM) for spectral information classification. The CNN-BiLSTM model offers robust feature-extraction capabilities while simultaneously considering the spectral reflectance sequence’s front and back band information for each sample point [30]. This fusion of CNN and BiLSTM allows for comprehensive analysis and improves the classification performance of hyperspectral data.

Nowadays, the application of deep-learning technology in the image segment has attracted extensive attention. Ronneberger et al. proposed a U-shaped network (U-Net) at the MICCAI conference in 2015 [31]. U-Net has distinct advantages, including its ability to achieve high accuracy with less training data and its fast training speed [32]. To extract the spatial relationships between pixels from hyperspectral images, this study trained the semantic segmentation model based on U-Net. The model trained with U-Net can restrain the irrelevant regions in an input image and highlight the striking features suitable for specific tasks [33]. It is conducive to eradicating the inevitability of applying backgrounds of cascading CNNs.

On this basis, the study developed a spatial–spectral combination algorithm wherein the majority category of pixels within a region determines the category assigned to the entire region. On the basis of U-Net for image segmentation, the CNN-BiLSTM model is used to recognize fragments and background pixel by pixel. The category with the highest number of pixels in each region represents the category of that region, accurately identifying the target fragments from the background. The method increases the precision of the model and retains the spatial data aimed at 2D segmentation. By combining both the spatial and spectral information, the method effectively leverages the strengths of the respective techniques.

The main technical contributions of this work are summarized as follows:

(1) Creation of datasets: We collected hyperspectral images containing five types of explosive fragments and five common background materials. We preprocessed these data, including black-and-white correction and region of interest (ROI) extraction. These datasets include both spectral and spatial information, providing valuable resources for further analysis;
(2) Spectral information classification model: To classify the spectral information accurately, we adopted the CNN-BiLSTM model. This novel approach combines CNN and BiLSTM to achieve high classification accuracy, even with limited training samples;
(3) Spatial–spectral combination algorithm: We designed a spatial–spectral combination algorithm that successfully classifies targets in hyperspectral images. By combining U-Net with CNN-BiLSTM, we effectively merge both spatial and spectral information, enhancing the overall classification performance;
(4) Experimental verification: To validate the effectiveness of our hyperspectral imaging detection methods in the identification of explosive fragments, we conducted classification experiments on the collected datasets. The results demonstrate the feasibility and potential of our approach.

This study used hyperspectral imaging technology for fragment classification based on the differences in explosion fragments and background spectral reflectance information. This research could effectively distinguish between fragments and background in a laboratory environment and visualize the materials and positions of different fragments and background materials. The purpose of this study is to verify the feasibility of using hyperspectral imaging to identify explosive fragments. At the same time, it provides guidance for the intelligent identification and recovery of fragments in the field in the future and has practical significance for evaluating the power of fragments.

## 2. Materials and Methods

### 2.1. Hyperspectral Imaging System

**Figure 1:**
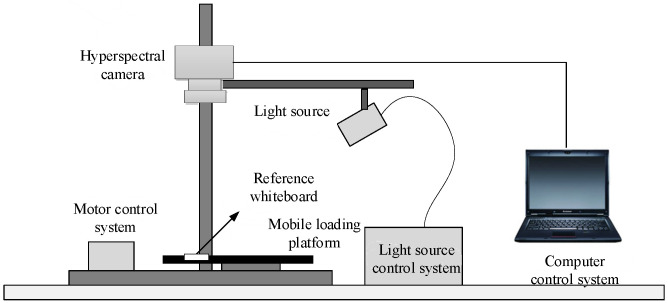
Schematic diagram of hyperspectral image acquisition system.

The hyperspectral camera model used in this study is a Hypersec VNIR-A (Headwall, Boston, MA, United States), which consists of a 1600×2500 high-resolution CCD lens, a total reflection grating, and a slit. The band interval for the sample data is 2.5 nm, and a total of 234 bands from 400 to 1000 nm are used for the spectral imaging of the experimental samples. The hyperspectral image acquisition system includes a hyperspectral camera, a light source, a light source control system, a reference whiteboard, a mobile loading platform, a motor control system, and a computer. The light source adopts a halogen lamp, which can meet the detector’s requirements for the wavelength range of the light source. The schematic diagram of the hyperspectral image acquisition system is shown in Figure 1. The specific parameters of the components of the hyperspectral imaging system used in this study are shown in Table 1.

**Figure 2:**
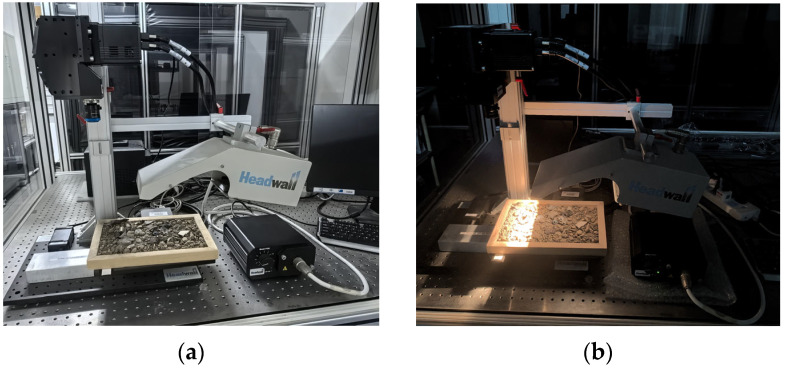
Hyperspectral image acquisition system. (**a**) Actual scene diagram; (**b**) darkroom environment diagram.

First, we performed image acquisition in an optical darkroom to reduce the impact of ambient light on the spectral information of the sample. We adjusted the brightness of the halogen light source to avoid image oversaturation. Second, we set the camera integration time to 40 ms and the gaze time to 39 ms to prevent image distortion. The distance between the test sample and the camera slit was 30 cm. Finally, we set the speed of the mobile loading platform to 2.168 mm/s to ensure that the collected sample images were clear and frame-accurate. Through these control measures, we could ensure that the data collected in the experiment met the experimental requirements and that the spectral and spatial information of the samples was complete and accurate. The actual scene image of the hyperspectral image acquisition system is shown in Figure 2a, and the darkroom environment image is shown in Figure 2b.

### 2.2. Black-and-White Board Correction

To reduce the image noises caused by the camera’s dark current and light, it is necessary to perform the black-and-white board correction on the collected hyperspectral images. We used a reference whiteboard to perform the black-and-white correction on the collected hyperspectral images. The correction formula is defined as:

(1) $$R_{norm}=\frac{R_{raw}-R_{dark}}{R_{white}-R_{dark}}$$

where $R_{norm}$ is the hyperspectral image after the black-and-white correction, $R_{raw}$ is the original hyperspectral image, $R_{dark}$ is the full dark image collected without removing the lens cover, and $R_{white}$ is the image obtained from a standard reference whiteboard with a reflectance of 99%.

### 2.3. Experiment Datasets

#### 2.3.1. Fragments and Background Samples

**Figure 3:**
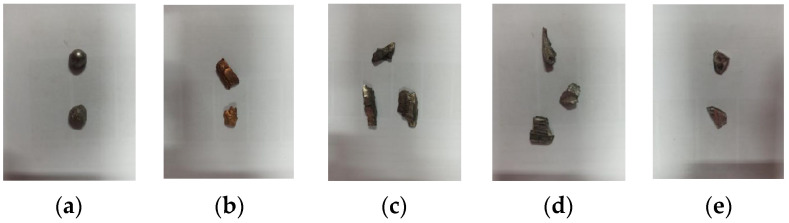

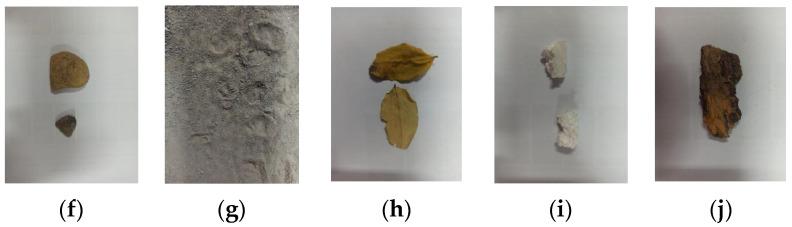
True-color images of the collected fragments and background samples. (**a**) Tungsten fragments; (**b**) copper fragments; (**c**) iron fragments; (**d**) aluminum fragments; (**e**) composite fragments; (**f**) stone samples; (**g**) soil samples; (**h**) leaf samples; (**i**) foam samples; (**j**) bark samples.

Generally, fragments are often made of different materials such as steel, synthetic materials, tungsten, copper, iron, and aluminum to achieve varying degrees of damage effects on different types of targets [34]. We collected five kinds of explosive fragments, as well as five common background materials. The true-color images of the collected fragments and background samples are shown in Figure 3.

The first line displays images (a) to (e), which depict tungsten fragments, copper fragments, iron fragments, aluminum fragments, and composite materials, respectively. The second line shows images (f) to (j), representing background materials, stones, soil, leaves, foams, and barks, respectively.

#### 2.3.2. Extracting Regions of Interest

**Figure 4:**
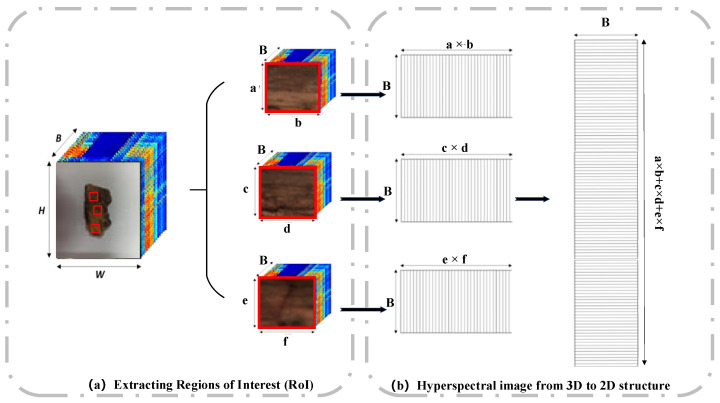
Region of interest extraction and data conversion of bark samples.

Extracting regions of interest (RoI) not only removes redundant data but also improves the speed of image feature processing and analysis and eliminates interference from other irrelevant data [35]. For hyperspectral remote-sensing images, the closed boundaries of the target area can be obtained by extracting interested objects, which can be used for subsequent object classification. Before performing dimensionality reduction, the input data needs to be converted from 3D to 2D. Region of interest extraction and data conversion of bark samples are illustrated in Figure 4.

In Figure 4, we captured a hyperspectral sample image using a hyperspectral system, with dimensions of length (H), width (W), and band number (B). Employing three rectangular boxes, sized a ×b, c × d, and e × f, we extracted the regions of interest, resulting in three distinct regions with dimensions of a × b × B, c × d × B, and e × f × B. Each 3D region of interest was converted into 2D hyperspectral data with respective lengths of a × b, c × d, e × f, and a width of B. Finally, all the data was concatenated along the length direction to form a 2D hyperspectral image data of (a × b + c × d + e × f) × B. In this experiment, we extracted regions of interest from ten collected samples, with 4000 sample points extracted from each sample. Finally, a total of 40,000 data points were used as the datasets of the spectral information. The dataset was divided into training sets, validation sets, and test sets in a 6:2:2 ratio through random sampling. The training set consisted of 24,000 sample points, while the validation and testing sets each contained 8000 sample points.

### 2.4. Convolutional Neural Network–Bidirectional Long Short-Term Memory Network

CNN-BiLSTM is the deep-learning model that combines CNN and LSTM [36]. It extracts features of different sizes through the convolution layers in CNN and effectively captures the contextual relationships in text using BiLSTM, thus endowing the model with strong feature-extraction and memory capabilities [37]. The model is mainly divided into the 1D-CNN part, the LSTM part, and the output part. The detailed process is as follows.

**Figure 5:**
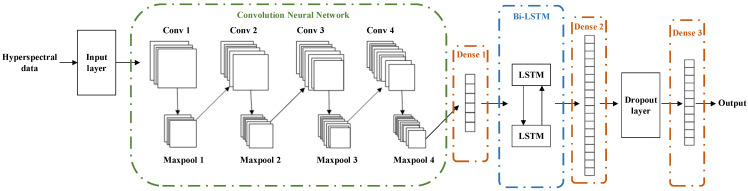
Model structure of CNN-BiLSTM.

During the training stage, the CNN component of the network employs a structure comprising four convolutional layers and four maxpooling layers. The features extracted by the CNN are then passed through a dense layer and fed into the BiLSTM network [38]. The BiLSTM component consists of two layers of LSTM networks, leveraging the distinct network structure of LSTM to further extract the time series features that may have been overlooked by the 1D-CNN component. This process enhances the accuracy of the classification model. The features extracted by the BiLSTM are then passed through a dense layer and fed into the dropout layer. Finally, the classification results are obtained through dense layers. The model structure is shown in Figure 5.

Due to the deep network structure employed in the aforementioned model, there exists a risk of overfitting during the training process. To mitigate this risk, a dropout layer has been included between dense layer 2 and dense layer 3. This layer serves the purpose of adjusting the weights of neurons and decreasing reliance on any specific neuron. Furthermore, the dropout layer aids in reducing the influence of noise and enhancing the model’s robustness. The loss function utilized in this model is the cross-entropy loss function, while the gradient descent optimization algorithm employs the Adam optimizer.

### 2.5. U-Shaped Network

**Figure 6:**
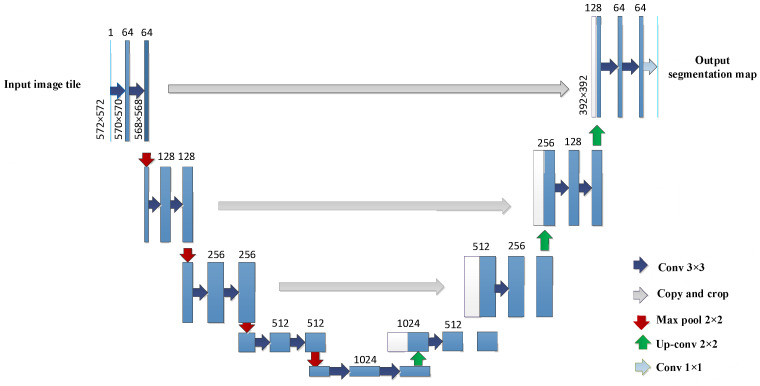
U-Net network structure diagram.

In order to obtain the spatial distribution pattern of fragments through a streamlined research process, traditional methods were anticipated to be substituted with artificial intelligence techniques to enhance the intelligence of this study. Therefore, this study adopted a semantic segmentation model based on U-Net to solve the problem of spatial region segmentation. U-Net is a neat end-to-end neural network architecture, which is known for its “U” shape [39]. The U-Net structure diagram is shown in Figure 6.

In the above figure, the numbers 1, 64, 128, 256, 512, and 1024 represent the number of image channels. The size of the input image is 572 × 572. Blue/white boxes represent feature maps. Blue arrows represent the 3 × 3 convolution for feature extraction. Grey arrows represent the skip connections for feature fusion. Red arrows indicate the maximum pooling, used to reduce dimensions. Green arrows indicate the up-convolution, used to restore dimensions. Baby-blue arrows represent the 1 × 1 convolution, used to output the results [31].

The compiler of U-Net consists of multiple convolutional and pooling layers. In this article, the convolutional kernel size used in the structure of U-Net is 3 × 3, with a step size of 1. The pooling layer adopts maximum pooling, which means that the pooled value of the area is the maximum value of the area. The size of each pooling layer kernel is 2. The padding is 0, and the step size is 2.

**Figure 7:**
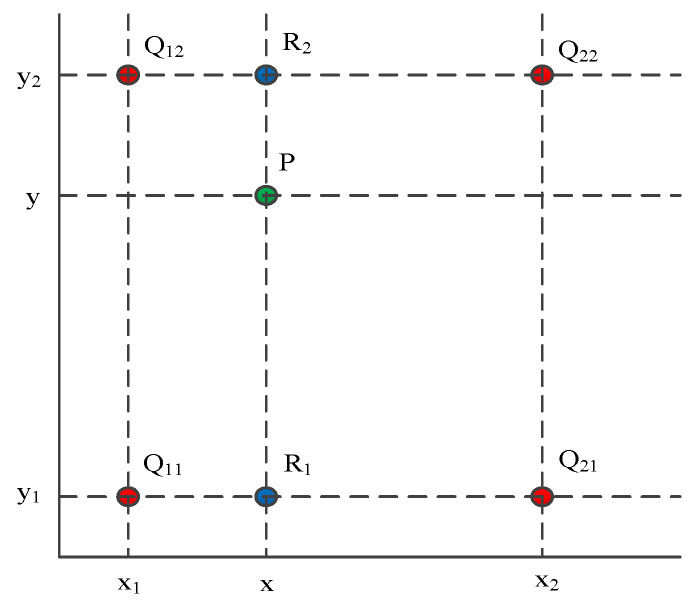
Up-sampling process based on bilinear interpolation.

A decoder is a process of restoring the original size of a feature map, which consists of convolution, up-sampling, and skip structures. U-Net uses bilinear interpolation for up-sampling, which has two independent variables. Linear interpolation is applied in both directions, using the original image matrix as the source matrix and the interpolated matrix as the target matrix. The up-sampling process, based on bilinear interpolation, is shown in Figure 7.

As shown in Figure 7, the horizontal and vertical axes represent the positions of the image pixels. $f\left(x,y\right)$ represents the color or grayscale value of the pixel at position $\left(x,y\right)$. We know the coordinates and corresponding pixel values of red dots $Q_{11}(x_{1},y_{1})$, $Q_{12}(x_{1},y_{2})$, $Q_{21}(x_{2},y_{1})$, $Q_{22}(x_{2},y_{2})$, $f\left(Q_{11}\right)$, $f\left(Q_{12}\right)$, $\;f\left(Q_{21}\right)$, and $f\left(Q_{22}\right)$. Our goal is to obtain the pixel values of the unknown function f at the green point P(x,y), which is $f\left(P\right)$. First, perform linear interpolation in the *x* direction to obtain $f\left(R_{1}\right)$ and $f\left(R_{2}\right)$. $f\left(R_{1}\right)$ is the value of blue point $R_{1}(x,y_{1})$ obtained by performing one-dimensional linear interpolation in the *x*-axis direction based on $f\left(Q_{11}\right)$, $f\left(Q_{12}\right)$. $f\left(R_{2}\right)$ is the value of blue point $R_{2}(x,y_{2})$ obtained by performing one-dimensional linear interpolation in the *x*-axis direction based on $f\left(Q_{21}\right)$, $f\left(Q_{22}\right)$.

(2) $$f(R_{1})\approx \frac{x_{2}-x}{x_{2}-x_{1}}f(Q_{11})+\frac{x-x_{1}}{x_{2}-x_{1}}f(Q_{21})$$

(3) $$f(R_{2})\approx \frac{x_{2}-x}{x_{2}-x_{1}}f(Q_{12})+\frac{x-x_{1}}{x_{2}-x_{1}}f(Q_{22})$$

Then, perform linear interpolation in the *y*-direction to obtain the $f\left(P\right)$ based on $f\left(R_{1}\right)$, $f\left(R_{2}\right)$.

(4) $$f(P)\approx \frac{y_{2}-y}{y_{2}-y_{1}}f(R_{1})+\frac{y-y_{1}}{y_{2}-y_{1}}f(R_{2})$$

Overall, the result of bilinear interpolation is $f\left(x,y\right)$:

(5) $$\begin{array}{l} f(x,y)\approx \frac{f(Q_{11})}{\left(x_{2}-x_{1})(y_{2}-y_{1}\right)}(x_{2}-x)(y_{2}-y)+\frac{f(Q_{21})}{\left(x_{2}-x_{1})(y_{2}-y_{1}\right)}(x-x_{1})(y_{2}-y)\\ +\frac{f(Q_{12})}{\left(x_{2}-x_{1})(y_{2}-y_{1}\right)}(x_{2}-x)(y-y_{1})+\frac{f(Q_{22})}{\left(x_{2}-x_{1})(y_{2}-y_{1}\right)}(x-x_{1})(y-y_{1}) \end{array}$$

In terms of skip structure, U-Net adopts the method of concatenating features from different levels in the channel dimension to form more “thick” features, which are the blue and white channels in Figure 5.

### 2.6. Spatial–Spectral Combination

**Figure 8:**
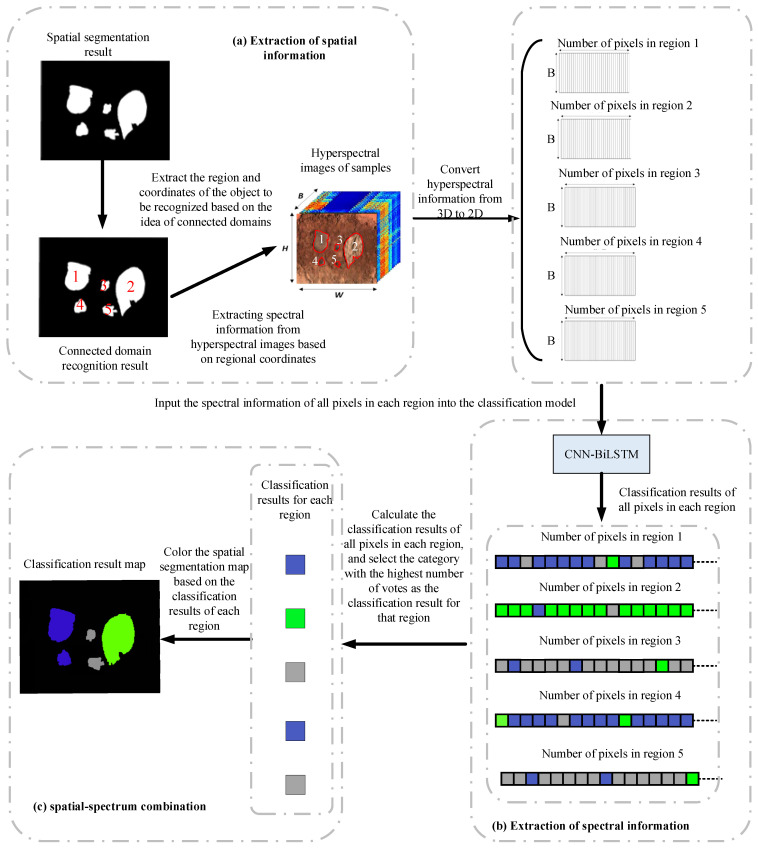
Flow chart of the spatial–spectrum combination.

Building upon previous research, this article utilized a spatial–spectral combination approach, leveraging the distinctive attributes of fragments and hyperspectral images. Following the spatial segmentation, the subsequent step involved identifying each segmented region using spectral information. This process enabled an accurate differentiation between the different types of targets and the background materials. The flow chart of the spatial–spectrum combination is shown in Figure 8.

This study obtained a binary image through U-Net segmentation, where black represented the background and white represented the target to be classified. We used the concept of connected domains to write algorithms that numbered all regions to be classified and extracted the coordinates of all pixels within each region. This created a mask based on these coordinates to cover the hyperspectral image and converted the 3D hyperspectral data of each region into 2D. Input the 2D hyperspectral data into a pre-trained CNN-BiLSTM model to classify each pixel in the region. By counting the categories of pixels within each region, the category of the region was determined by the categories of the majority of pixels within that region. Then, the region was colored based on the different classification results to obtain a spatial map of the fragment classification results.

### 2.7. Semantic Segmentation Datasets

**Figure 9:**
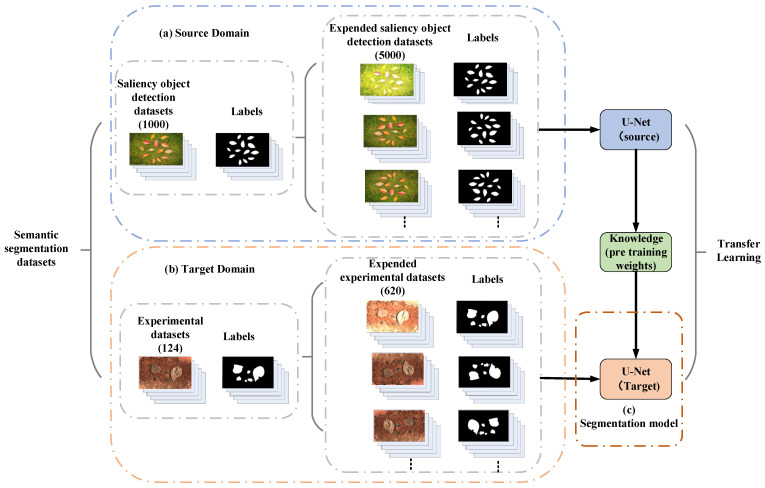
Composition of semantic segmentation dataset based on transfer learning.

To obtain the spatial distribution of fragments, this study employed a semantic segmentation model based on U-Net to accurately segment targets in explosive fragment dispersion fields. However, due to the limited number of experimental samples, it was challenging to train a semantic segmentation model with sufficient generalization ability. To address this issue, the study adopted the concept of transfer learning and utilized training datasets from two sources. The first part of the training dataset comprised 1000 images from the saliency object detection datasets that met the required criteria. The second part consisted of 124 images collected from laboratory experiments and online sources. Additionally, the study incorporated an extended dataset specifically created for this research. This dataset was expanded by applying various transformations, such as rotation, brightness adjustment, cropping, and other methods, resulting in a collection of 5620 images. The composition of the semantic segmentation dataset based on transfer learning is shown in Figure 9. These images were calibrated in the image label software included in MATLAB 2019b.

### 2.8. Hyperspectral Image of the Dispersion of Fragments

**Figure 10:**
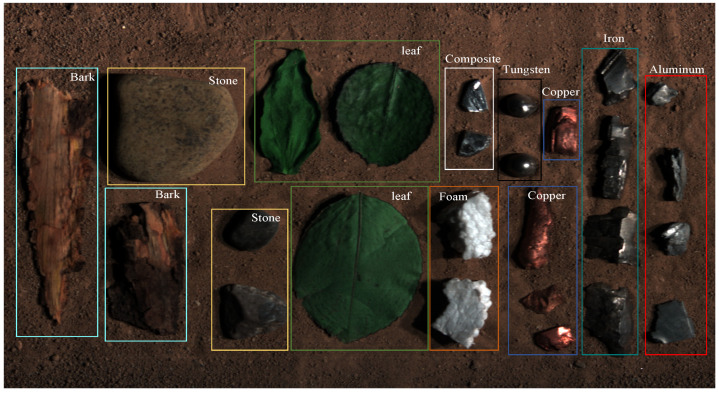
Hyperspectral image of the dispersion of fragments.

The spatial distribution and spectral data of the fragments could be obtained from the hyperspectral images of the dispersion of fragments. The hyperspectral image of the dispersion of fragments is shown in Figure 10.

### 2.9. Construction of Classifying Explosive Fragments

**Figure 11:**
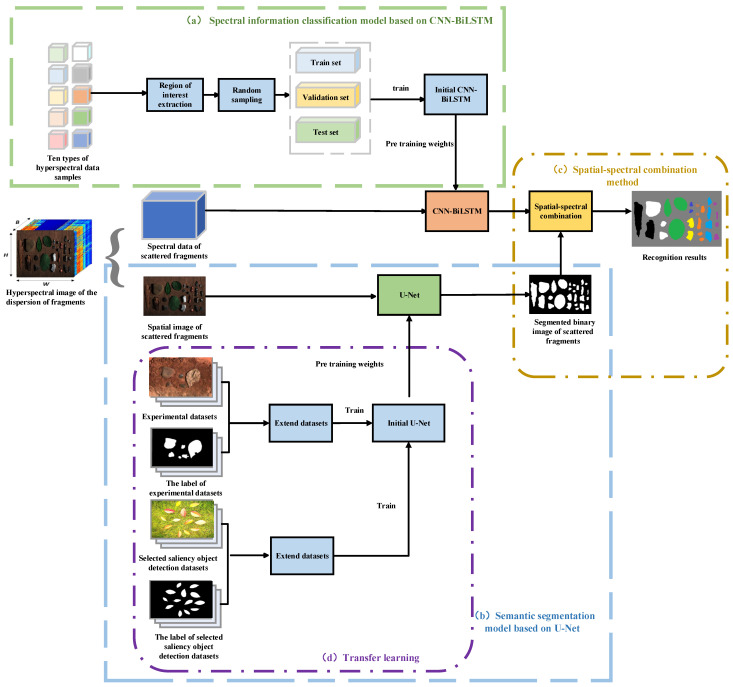
Overall procedure of the proposed classification of hyperspectral images of the explosive fragments framework including data augmentation, spectral information classification model based on CNN-BiLSTM, semantic segmentation model based on U-Net, and spatial–spectral combination method.

The overall procedure of the proposed framework for classifying explosive fragments is depicted in Figure 11. First, the spectral information classification model based on CNN-BiLSTM was trained. Ten types of hyperspectral data samples were collected in a laboratory environment, and RoIs were extracted from each sample type, resulting in 4000 sample points per type. These datasets were randomly divided into training sets, validation sets, and test sets. The training sets had 24,000 sample points, and the validation sets and testing sets both had 8000 sample points. The input data were fed into the CNN-BiLSTM model, and network parameters were adjusted to achieve the desired classification accuracy. The main parameters of the CNN-BiLSTM model are shown in Table 2. Second, a semantic segmentation model based on U-Net was trained. The U-Net model was trained using an extended dataset that included saliency object detection datasets, as well as images collected from laboratory experiments. We input the spatial image of scattered fragments to obtain the segmented binary image of the scattered fragments. The main parameters of the U-Net model are shown in Table 3. Finally, we input the spectral data of scattered fragments into the trained CNN-BiLSTM model to obtain the classification results. Then, the classification results were combined with the segmented binary image, where the category of the region was determined by the majority of pixels within that region.

From Table 2, 24,000 training sets were divided into 240 subsets, and every subset contains 100 samples. These subsets were called mini-batches, and the mini-batch size of the network was 100. During the training process of each subset, a gradient descent was performed, and 240 iterations were performed. The behavior of traversing all samples once was called an epoch, and the total epochs of the model were 80. An Adam loss function was selected to update the model parameters as the model optimizer. The number of filters was 20, and the size of the filter was 5*5. The number of hidden units was 100, which allowed the network to have strong computing power while preventing overfitting. Due to the experiment datasets having a total of 10 types of samples, the number of classes in the model was set to 10.

In Table 3, we aimed to segment both the background and target, constituting a total of 2 classes. The input image dimensions were 512 × 512 pixels. Each batch consisted of 5 samples, with training extending 50 epochs. The initial learning rate was set at 0.001, and the model optimization was handled by the Adam optimizer. When the momentum parameter was excessively high, it failed to effectively capture the overall data trend. Conversely, if it was too low, the model could overfit, leading to substantial fluctuations between adjacent data points. A momentum value of 0.9 struck a balance, enabling an accurate reflection of the data trend without excessive oscillations. In practice, momentum values around 0.9 were commonly employed. Throughout the training, the model weights and corresponding loss function values were periodically saved every five iterations. This approach facilitated monitoring changes in the loss function and segmentation accuracy over the training course, with a save interval of 5 in the model structure. Following the 235th training iteration, the model achieved its lowest loss value of 0.025, and the weights at this point were fed into the U-Net architecture. The training speed heavily relied on the number of workers, influencing data loading efficiency, which had been set at 4 in that scenario. The dataset comprised 3264 training samples and 816 validation samples, crucial for model evaluation and performance assessment.

In order to reduce the influence of random factors, all experiments were repeated ten times, and the average value was taken as the final experimental result. The running environment of the experimental code in this article is as follows. The CPU was a 13th Gen Intel(R) Core (TM) i9-13900K. The primary frequency was 3.00 GHz. The memory capacity was 128 GB. The GPU was an NVIDIA GeForce GTX 3090. The memory was 64 GB. The programming language was Python3.11, and the deep-learning architecture was PyTorch1.10.0.

## 3. Experimental Results and Analysis

### 3.1. Spectral Curve Analysis

**Figure 12:**
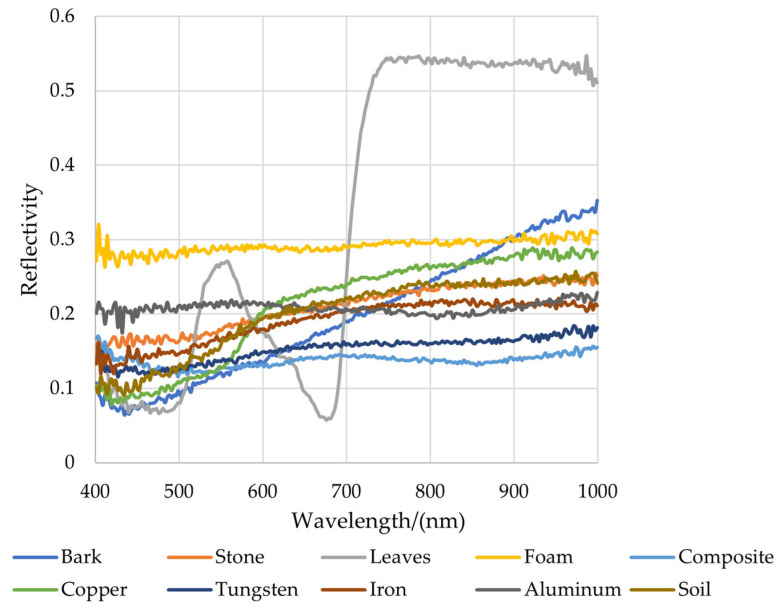
Full-band reference spectral curve of samples.

There are 50 randomly selected representative sample points from the RoIs of each type of sample. The calculated average values of the spectral information for each category and obtained the spectral reflectance curve are shown in Figure 12.

From Figure 12, it could be seen that only the reflectance of the leaves had a significant difference compared to other species. In the visible light spectrum, chlorophyll was the main factor governing plant spectral response. Chlorophyll absorbed most of the incident energy in two spectral bands, with central wavelengths of 0.45 μm (blue) and 0.67 μm (red), respectively. Between these two chlorophyll absorption bands, chlorophyll had a relatively small absorption effect around 0.54 μm (green), forming a reflection peak, so our eyes saw the plant as green. In the near-infrared band, the spectral characteristics of vegetation were mainly influenced by the internal structure of the plant leaves. The spectral characteristic of healthy green plants in the near-infrared band was high reflectivity. Between the visible and near-infrared bands, around 0.76 μm, the reflectivity sharply increased, forming the so-called “red edge”, which was the most obvious feature of the plant curve. There were also certain differences in reflection intensity or overall trend for different fragments and other types of background materials. We could establish classification models for different fragments and background materials based on these differences.

### 3.2. Spectral Information Classification Model Results and Analysis

In this study, the classification accuracy was used as the evaluation metric for the model. The overall accuracy (OA) and average accuracy (AA) were used to evaluate the classification performance [40]. The OA is calculated as the number of samples predicted correctly divided by the total samples of the test set, as shown in Formula (6). AA is the ratio of the sum of classification accuracy for each class to the number of classes, as shown in Formula (7). The precision, recall, and F1 score are shown in Formulas (8)–(10), respectively. The above evaluation indicators are dimensionless. The range of these parameters is from zero to one, where one represents the best performance and zero represents the worst performance.

(6) $$\mathrm{Overall}\;\mathrm{Accuracy}=\frac{TP+TN}{FN+TP+FP+TN}$$

(7) $$\mathrm{Average}\;\mathrm{Accuracy}=\frac{\mathrm{Overall}\;\mathrm{Accuracy}}{N}$$

(8) $$\mathrm{Precision}=\frac{TP}{TP+FP}$$

(9) $$\mathrm{Recall}=\frac{TP}{FN+TP}$$

(10) $$F1=\frac{2\times Precision\times Recall}{Precision+Recall}$$

*TP* denotes the number of samples labeled as positive and classified as positive. *FP* denotes the number of samples labeled as negative and classified as positive. *TN* denotes the number of samples labeled as negative and classified as negative. *FN* denotes the number of samples labeled as positive and classified as negative. *N* is the number of classes. Because the above variables represent different types of sample sizes, their dimensions are “units”.

In order to demonstrate the good performance of CNN-BiLSTM in fragment hyperspectral image classification, the experiment used decision trees, support vector machines (SVM), CNN, BiLSTM, and CNN-BiLSTM to classify 8000 samples in the test set. The confusion matrix was a visualization method that displays the performance of an algorithm in a specific matrix [41]. Each column in the matrix represents the predicted label, and each row represents the actual label. Since the number of samples in each category is 800, the OA is equal to the AA.

**Figure 13:**
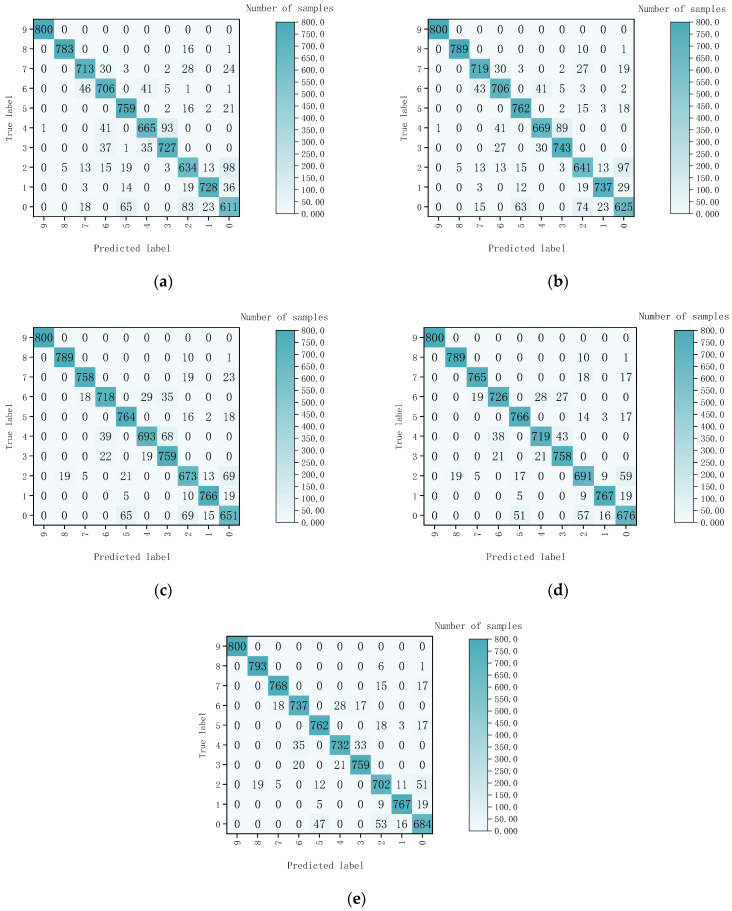
Confusion matrix results of the five-class classification models. (**a**) Decision tree; (**b**) SVM; (**c**) CNN; (**d**) BiLSTM; (**e**) CNN-BiLSTM.

The confusion matrix results of the five-class classification models are shown in Figure 13. Labels 0, 1, 2, 3, 4, 5, 6, 7, 8, and 9, respectively, represent tungsten fragments, aluminum fragments, iron fragments, copper fragments, composite fragments, bark, soil, stone, foam, and leaves.

From Figure 13, the misjudgment rate between different types of fragments and background materials was relatively low. The classification between the fragments had a higher misjudgment rate, such as tungsten fragments and iron fragments. This finding indicated that distinguishing between non-metals was easier to achieve than between metals. Finally, the recognition rate of each class of samples and the AA are shown in Table 4. Bold numbers represent the highest accuracy.

According to Table 4, the accuracies of CNN, BiLSTM, and CNN-BiLSTM were significantly higher than that of decision trees and SVM. This result indicated that deep learning had significantly improved classification accuracy. The average accuracy of the CNN BiLSTM classification model reached 93.8%, surpassing decision trees, SVM, CNN, and BiLSTM. The CNN-BiLSTM model was only slightly inferior to the BiLSTM in bark sample classification and had the highest classification accuracy for other categories of samples. Compared to using CNN and BiLSTM separately, the CNN-BiLSTM model was more suitable for the spectral information classification of fragments. Among the three classification models, the classification accuracies for leaves and foam were particularly high, more than 99%. On the other hand, the classification accuracy of tungsten fragments and iron fragments was relatively low, but still above 80%. The precision, recall, and F1 scores of the different classification models are shown in Table 5.

### 3.3. Semantic Segmentation Results and Analysis

**Figure 14:**
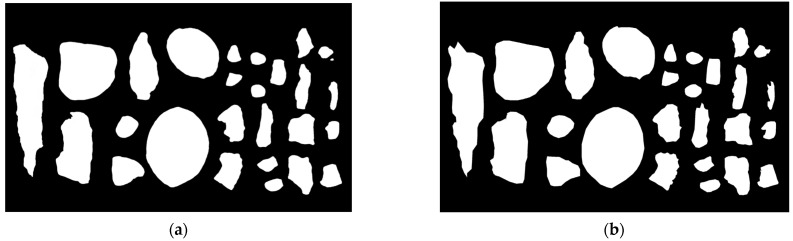
Semantic segmentation results and calibration results. (**a**) Segmentation results; (**b**) calibration results.

The model segmented hyperspectral spatial images with fragments scattered in the background, where the white part represented the target to be classified. The results are shown in Figure 14, where (a) was the segmentation map and (b) was the calibration map.

The intersection over union (*IoU* and true positivity ratio (*TPR*) were used to evaluate the semantic segmentation results. The *IoU* and *TPR* are dimensionless. The range of these parameters is from zero to one, where one represents the best performance and zero represents the worst performance. The calculation formulas are as follows.

(11) $$IoU=\frac{TP}{FN+TP+FP}$$

(12) $$TPR=\frac{TP}{TP+FN}$$

*TP* (true positive) is the intersection of the segmentation results and the calibration results. *FP* (false positive) is the over-segmentation part. *FN* (false negative) is the under-segmentation part [42]. Because the above variables represent different types of sample sizes, their dimensions are “units”. The evaluation results are shown in Table 6.

Since the *IoU* and *TPR* of this experiment were both more than 0.9, it indicated that the spatial segmentation method based on U-Net had achieved good results in the images.

### 3.4. Identification Results and Analysis of Explosive Fragments Based on the Spatial–Spectral Combination

**Figure 15:**
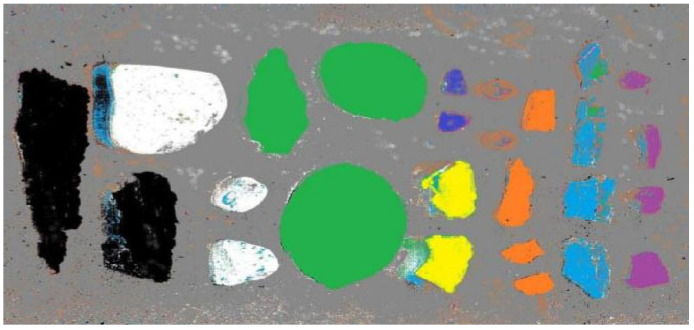
Classification results of CNN-BiLSTM model.

After training on spectral reflectance classification, three deep-learning classification models were developed. Among these models, the CNN-BiLSTM model exhibited the highest classification accuracy. Consequently, this model was employed to classify the hyperspectral data of scattered fragments on a pixel-by-pixel basis. The resulting classification outcomes are presented in Figure 15.

In Figure 15, the different colors of the pixels represent various materials. Specifically, gray represented sand, black represented bark, green represented stones, yellow represented leaves, dark blue represented foam, brown represented composite fragments, orange represented tungsten fragments, blue represented aluminum fragments, and purple represented copper fragments.

**Figure 16:**
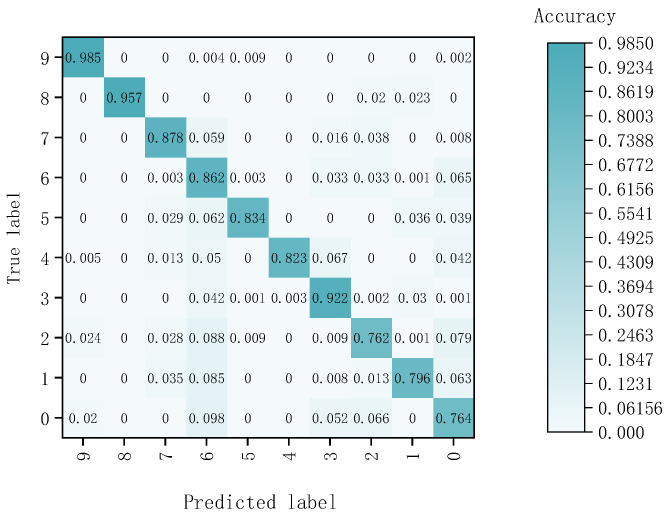
Confusion matrix results of the CNN-BiLSTM models.

Utilizing semantic segmentation, the classification results obtained from the CNN-BiLSTM model were further analyzed by counting the pixels in each region. The confusion matrix results of the CNN-BiLSTM models are shown in Figure 16. Labels 0, 1, 2, 3, 4, 5, 6, 7, 8, and 9, respectively, represented tungsten fragments, aluminum fragments, iron fragments, copper fragments, composite fragments, bark, soil, stone, foam, and leaves. The recognition of each sample class based on the CNN-BiLSTM model is shown in Table 7, and the results of the evaluation metrics based on CNN-BiLSTM are shown in Table 8.

**Figure 17:**
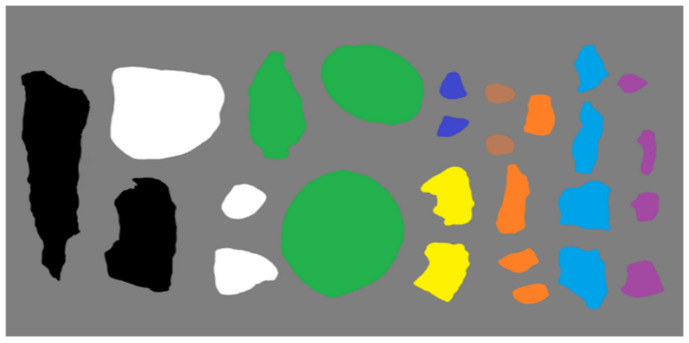
Results of spatial–spectral combination.

The OA and AA of the samples were determined to be 87.2% and 85.8%, respectively. Among the different sample types, leaves exhibited the highest accuracy at 98.5%, while iron fragments had the lowest recognition accuracy at 76.2%. It should be noted that the misclassified pixels were primarily located at the edges, regions that were excessively bright or dark, and areas affected by shadows resulting from the angle of light. The number of different sample points varied in hyperspectral images. The number of pixels in the soil samples and bark samples was high, while the number of pixels in the aluminum fragment samples, iron fragment samples, and copper fragment samples was low. The reason for the low precision and F1 scores of aluminum fragments was that soil samples were misclassified as aluminum fragment samples. The reason for the low precision and F1 scores of iron and copper fragments was that the bark samples were misclassified into iron and copper fragments. Subsequently, the classification results based on the CNN-BiLSTM model were combined with the spatial segmentation map generated by U-Net. Clustering was performed based on the segmented areas, with the type of each area determined by the dominant pixel category. The recognition result is shown in Figure 17.

The ratio of the intersection points of algorithm segmentation and manual calibration to the calibration results was used to evaluate the final recognition results. The spatial–spectral combination recognition rate and the result of the evaluation index are shown in Table 9.

From Table 9, the recognition rate of all samples was higher than 94%, and the OA and AA of the samples were 96.4% and 96.3%, respectively. It showed a significant improvement compared to the recognition rate in the spectral domain.

## 4. Discussion

This study used hyperspectral imaging technology for fragments classification based on the differences in explosion fragments and their background spectral reflectance information. We collected ten samples of tungsten fragments, iron fragments, copper fragments, aluminum fragments, composite fragments, soil, stones, leaves, bark, and foam. In a darkroom environment, full-band hyperspectral images in the range of 400.00 to 1000.00 nm of ten types of samples and fragment simulation scattering scenes were collected. After preprocessing, a deep-learning method was used to build a spatial–spectral combination approach for fragment classification using hyperspectral imaging, aiming to address the requirements of fragment identification and spatial dispersion analysis in ex-plosive fields. In addition, some further discussions are presented below.

In our previous research, there was a problem of low accuracy in using decision trees to identify stones and iron fragments [19]. This was because the decision tree model did not extract appropriate features, and the model was susceptible to noise and outliers. From Table 4, we can see that the classification accuracy of CNN, BiLSTM, and CNN-BiLSTM based on deep learning is significantly higher than that of decision tree and SVM. This was because deep-learning algorithms can automatically capture the correlation between spectral features and obtain the intrinsic characteristics of explosive fragments. The accuracy of CNN was slightly lower than that of BiLSTM. The reason for this situation is that the spectral information of sampling points in hyperspectral images is typical sequential data. The BiLSTM is better at handling sequence-type data than the CNN. The CNN-BiLSTM combined the advantages of CNN and BiLSTM, with an accuracy rate of up to 93.8%. It proved that the method was beneficial for identifying explosive fragments using hyperspectral images.

In the application of hyperspectral image segmentation, most studies only treated segmentation as a part of preprocessing and did not delve into it in-depth [43]. Currently, most studies use machine-learning-based segmentation methods [44,45]. The segmentation method we used in our previous research was based on morphology [19]. The two methods required human involvement to some extent. Therefore, we trained a U-Net model that could automatically extract explosion fragment features for fragment and background object segmentation using the idea of transfer learning. The trained U-Net network achieved an accuracy of 93.5% in segmenting sample images. The results indicated that U-Net could effectively segment fragments in the background from other background objects and extract the spatial features of the samples.

This study used the spatial–spectral combination method to identify explosive fragments, which could classify and express targets in the scattering field, with a recognition rate of better than 94.9% for each category. Compared to using the CNN-BiLSTM model to classify each pixel in the scattering field, the spatial–spectral combination algorithm significantly improved the fragment recognition rate. Compared with previous studies, we found that the algorithm used in this article greatly reduced human involvement [19]. In recent years, many studies have focused on how to fully utilize the spatial and spectral information contained in hyperspectral images and have also proposed many novel algorithms, such as the random patches network (RPNet) [46], etc. However, these algorithms are usually used in publicly available hyperspectral datasets and are rarely applied in practical fields. We will study relevant algorithms in the future and truly apply novel algorithms to the field of explosive fragment recognition.

As above, the fragments classification method used in this article could automatically extract features in both the spatial and spectral domains and perform spatial–spectral combinations through a cascading approach. After experimental verification, it was feasible to use the method of spatial–spectral combination with hyperspectral imaging technology for the classification of explosive fragments.

## 5. Conclusions

This paper introduces a spatial–spectral combination approach for fragment classification using hyperspectral imaging, aiming to address the requirements of fragment identification and spatial dispersion analysis in explosive fields. Our work can be divided into three main aspects.

(1) This study used a hyperspectral imaging system to collect data on fragments and backgrounds. After denoising, the regions of interest were extracted and sample points were randomly selected in each region as the spectral information dataset for this project. Random sampling is used to divide the sample points into a training set, a validation set, and a testing set. By observing the spectral reflectance curves of explosion fragments and background samples, it can be found that there are spectral band differences in the spectral curves of different types of samples. A semantic segmentation training dataset was collected and established based on the characteristics of the simulated explosion fragment dispersion field in this study. Finally, we expanded and calibrated the spatial segmentation dataset;
(2) We address the limitation of CNN in considering the relationship between current data and its neighboring data, which significantly impacts classification accuracy. To overcome this, this study adopts the CNN-BiLSTM model as the spectral information classification model. The experimental results demonstrate the ability of CNN-BiLSTM to leverage the advantages of both CNN and BiLSTM, resulting in high accuracy for hyperspectral image classification even with a limited number of training samples. To further enhance the model’s performance, dropout layers are introduced to mitigate overfitting. In addition, we incorporate the U-Net semantic segmentation model to extract spatial relationships between pixels from hyperspectral images. This approach enables the precise segmentation of spatial images containing fragments, achieving high accuracy in the process;
(3) A classification framework based on the spatial–spectral combination is proposed. In this way, U-Net is combined with CNN-BiLSTM, which can fully merge the spatial and spectral information of hyperspectral images to achieve high classification accuracy. The experimental results demonstrate that the accuracy of fragment recognition based on the spatial–spectral combination is substantially higher compared to the accuracy achieved through spectral-based recognition alone. It indicates the feasibility of using hyperspectral detection methods for fragment recognition based on the spatial–spectral combination methods, providing an experimental and theoretical basis for the recognition of fragments.

The purpose of this study is to verify the feasibility of using hyperspectral imaging to identify explosive fragments. The relevant algorithms are currently only applicable for explosive fragment recognition in laboratory darkroom environments. We will test the accuracy of recognition in outdoor environments in the future. In addition, in our next research, we will identify different environments and unique features of fragments by combining multidimensional data. Due to the wide range of fragments scattered at the scene after the explosion, we will use airborne hyperspectral imaging to image the explosion fragments in the future. Considering the challenges posed by the complex light source and small fragment volume at the explosion site, our research will delve into spectral information correction models and explore more fitting classification algorithms. Due to the possibility of fragments being obstructed by soil or vegetation, we will apply more imaging methods, such as thermal infrared imaging and synthetic aperture radar (SAR) imaging with hyperspectral imaging, for image fusion in explosive fragment recognition in the future.

## Figures and Tables

**Table 1:** Main parameters of experimental system.

System Composition	Parameter	Values
Hyperspectral camera	Spectral range	400–1000 nm
	Spectral channels	923
	Spatial channels	1600
	Spectral resolution	2.5 nm
	Pixel size	6.5 μm
	Slit width	25 μm
	Maximum frame rate	100 fps
	Detector Type	CCD
Lens	Focal length	23 mm
	Field of view	25°
Halogen lamp	Power	150 W (adjustable)
	Illumination	Concave aluminum surface reflection
Reference whiteboard	Reflectivity	>99%
	Material	Polytetrafluoroethylene (PTFE)
Mobile loading platform	Length of rail	250 mm
	Step precision	0.5 μm

**Table 2:** Main parameters of spectral information classification model based on CNN-BiLSTM.

Main Parameter	Values
Mini Batch Size	100
Number of Hidden Units	100
Filter Size	5
Number of Filters	20
Epochs	80
Optimizer type	Adam
Number of classes	10
Number of test sets	8000
Number of train sets	24,000
Number of validation sets	24,000

**Table 3:** Main parameters of U-Net training model.

Main Parameter	Values
Num classes	2
Input shape	(512, 512)
Epoch	50
Batch size	5
Initial learning rate	0.0001
Optimizer type	Adam
momentum	0.9
Save period	5
Number of workers	4
Number of train sets	3264
Number of validation sets	816

**Table 4:** Accuracy statistics of different model classification test sets.

Model	Accuracy, %										
	Tungsten	Aluminum	Iron	Copper	Composite	Bark	Soil	Stone	Foam	Leaves	Average
Decision Tree	76.4	91.0	79.3	90.9	83.1	94.9	88.3	89.1	97.9	100	89.1
SVM	78.1	92.1	80.1	92.9	83.7	95.3	88.3	89.9	98.7	100	89.9
CNN	81.4	95.8	84.1	94.9	86.6	95.5	89.8	94.8	98.7	100	92.1
BiLSTM	84.5	**95.9**	86.4	94.8	89.9	**95.8**	90.8	95.6	98.7	100	93.2
CNN-BiLSTM	**85.6**	**95.9**	**87.8**	**94.9**	**91.5**	95.3	**92.1**	**96.0**	**99.1**	**100**	**93.8**

**Table 5:** Precision, recall, and F1 scores for different classification models.

Model	Index	Tungsten	Aluminum	Iron	Copper	Composite	Bark	Soil	Stone	Foam	Leaves
Decision Tree	Precision	0.771	0.950	0.795	0.874	0.897	0.882	0.852	0.899	0.994	1.000
	Recall	0.764	0.910	0.793	0.909	0.831	0.949	0.883	0.891	0.979	1.000
	F1 score	0.767	0.930	0.794	0.891	0.863	0.914	0.867	0.895	0.986	1.000
SVM	Precision	0.790	0.950	0.812	0.880	0.904	0.891	0.864	0.907	0.994	1.000
	Recall	0.781	0.921	0.801	0.929	0.836	0.953	0.883	0.899	0.986	1.000
	F1 score	0.785	0.935	0.806	0.904	0.869	0.921	0.873	0.903	0.990	1.000
CNN	Precision	0.834	0.962	0.844	0.811	0.935	0.894	0.922	0.971	0.976	1.000
	Recall	0.814	0.958	0.841	0.949	0.866	0.955	0.898	0.948	0.986	1.000
	F1 score	0.824	0.960	0.842	0.875	0.899	0.923	0.910	0.959	0.981	1.000
BiLSTM	Precision	0.857	0.965	0.865	0.915	0.936	0.913	0.925	0.970	0.976	1.000
	Recall	0.845	0.959	0.864	0.948	0.899	0.958	0.908	0.956	0.986	1.000
	F1 score	0.851	0.962	0.864	0.931	0.917	0.935	0.916	0.963	0.981	1.000
CNN-BiLSTM	Precision	0.867	0.962	0.874	0.938	0.937	0.923	0.931	0.971	0.977	1.000
	Recall	0.855	0.959	0.878	0.949	0.915	0.953	0.921	0.960	0.991	1.000
	F1 score	0.861	0.960	0.867	0.943	0.926	0.938	0.926	0.965	0.984	1.000

**Table 6:** Semantic segmentation results.

Evaluation Index	*IoU*	*TPR*
Values	0.935	0.974

**Table 7:** Classification accuracy of the CNN-BiLSTM models.

Sample	Size	Sample Point Classification										Accuracy, %
		Tungsten	Aluminum	Iron	Copper	Composite	Bark	Soil	Stone	Foam	Leaves	
Tungsten	19,096	14,589	0	1254	988	0	0	1874	0	0	391	76.4
Aluminum	46,835	2958	37,262	612	359	0	0	3985	1659	0	0	79.6
Iron	131,201	10,398	125	100,034	1235	0	1156	11,506	3623	0	3124	76.2
Copper	71,814	98	2124	112	66,204	187	84	3005	0	0	0	92.2
Composite	19,909	833	0	0	1342	16,384	0	997	251	0	102	82.3
Bark	306,003	11,984	10,897	0	0	0	255,247	18,875	9000	0	0	83.4
Soil	2,902,425	188,976	3371	95,894	94,685	206	7489	2,502,160	8672	531	441	86.2
Stone	234,432	1892	0	9002	3848	0	0	138,98	205,792	0	0	87.8
Foam	83,595	0	1928	1688	0	0	0	0	0	79,979	0	95.7
Leaves	424,668	845	0	0	0	0	3943	1765	0	0	418,124	98.5

**Table 8:** Evaluation index of CNN-BiLSTM.

Index	Tungsten	Aluminum	Iron	Copper	Composite	Bark	Soil	Stone	Foam	Leaves
Precision	0.813	0.669	0.480	0.561	0.977	0.953	0.978	0.899	0.993	0.990
Recall	0.764	0.796	0.762	0.922	0.823	0.834	0.862	0.878	0.957	0.985
F1 score	0.788	0.727	0.589	0.698	0.893	0.890	0.916	0.888	0.975	0.987
OA, %	87.2									
AA, %	85.8									

**Table 9:** Statistical table of the results of the spatial–spectral combination.

Samples	Tungsten	Aluminum	Iron	Copper	Composite	Bark	Soil	Stone	Foam	Leaves
Test sample points	19,096	46,835	131,201	71,814	19,909	306,003	2,902,425	234,432	83,595	424,668
Identify points	18,590	45,274	126,149	69,163	18,993	298,427	2,795,035	222,461	79,567	415,112
Accuracy, %	97.4	96.7	96.1	96.3	95.4	97.5	96.3	94.9	95.2	97.5
OA, %	96.4									
AA, %	96.3									

## Data Availability

The data are not publicly available due to the confidentiality of the research projects.

## Abbreviations

The following abbreviations are used in this manuscript:

CNN-BiLSTM	Convolutional Neural Network–Bidirectional Long Short-Term Memory Network
U-Net	U-shaped Network
CNN	Convolutional Neural Network
1D	One Dimensional
RNN	Recurrent Neural Network
BiLSTM	Bidirectional Long Short-Term Memory Network
RoI	Regions of Interest
OA	Overall Accuracy
AA	Average Accuracy
SVM	Support Vector Machines
IoU	Intersection over Union
TPR	True Positivity Ratio
TP	True Positive
FP	False Positive
FN	False Negative
RPNet	Random Patches Network
FSKNet	Faster Selective Kernel Mechanism Network
SAR	Synthetic Aperture Radar
